# The risk of revision surgery after trainee-led primary total hip replacement

**DOI:** 10.1308/rcsann.2024.0049

**Published:** 2024-11-21

**Authors:** DJ Howgate, P Garfjeld Roberts, A Palmer, A Price, A Taylor, JL Rees, B Kendrick

**Affiliations:** ^1^ University of Oxford, UK; ^2^ NIHR Oxford Biomedical Research Centre, UK; ^3^ Oxford University Hospitals NHS Foundation Trust, UK

**Keywords:** Hip, Total Hip Replacement, Education, Training, National Joint Registry

## Abstract

**Introduction:**

The aim of this study was to determine the impact of operating surgeon grade and level of supervision on the incidence of one-year patient mortality and all-cause revision following elective primary total hip replacement (THR).

**Methods:**

National Joint Registry data from 2005 to 2020 for a single University Teaching Hospital were used, with analysis performed on the 15-year dataset divided into 5-year block periods (B1, 2005–2010; B2, 2010–2015; B3, 2015–2020). Outcome measures were mortality and revision surgery at one year, in relation to lead surgeon grade, and level of supervision for trainee-led (TL) operations.

**Results:**

A total of 9,999 eligible primary THRs were performed, of which 5,526 (55.3%) were consultant-led (CL), and 4,473 (44.7%) TL. Of TL, 2,404 (53.7%) were nonconsultant-supervised (TU) and 2,069 (46.3%) consultant-supervised (TS). The incidence of one-year patient mortality was 2.05% (*n*=205), and all-cause revision was 1.11% (*n*=111). There was no difference in one-year mortality between TL and CL operations (*p*=0.20, odds ratio (OR) 0.78, confidence interval (CI) 0.55–1.10). The incidence of one-year revision was not different for TL and CL operations (*p*=0.15, OR 1.37, CI 0.89–2.09). Overall, there was no temporal change for either outcome measure between TL or CL operations. A significant increase in revision within one-year was observed in B3 between TU compared with CL operations (*p*=0.005, OR 2.81, CI 1.35–5.87).

**Conclusions:**

We found no difference in overall one-year mortality or all-cause revision rate between TL and CL primary THR. Despite a reduction in unsupervised THR in the latest five-year period (2015–2020), unsupervised TL THR resulted in an increased risk of early revision.

## Introduction

Trainees in orthopaedic surgery must obtain level 4 procedure-based assessment (PBA) in several indicative procedures to achieve a certificate of completion of training (CCT). Among the indicative procedures is total hip replacement (THR) – a common procedure requiring both generic surgical and procedure-specific skills. It is common for trainees to spend a minimum of 12 months in posts specifically to obtain training in lower limb arthroplasty to obtain the required volume and competency. However, training has changed significantly over the last 15 years, with reduced working hours and an increase in trainee numbers. Trainees subsequently have reduced access to common procedures. A recent publication from the National Joint Registry (NJR) regarding the impact of training on revision rates in THR showed that less than 10% of THRs are performed by trainees.^1^ This situation is compounded by surgeons' concerns of becoming an outlier for revision in NJR data, thus further reducing the number of procedures trainees are allowed to perform. There is also the potential negative impact of trainee involvement on the incidence of postoperative complications.^1–4^ The primary aim of this study was to investigate the impact of lead surgeon grade on the incidence within one year of potentially avoidable serious adverse patient outcomes following primary elective THR (mortality and revision). Secondary aims included defining any temporal changes in these outcome measures and indications for performing early revisions.

## Methods

**Figure 1:**
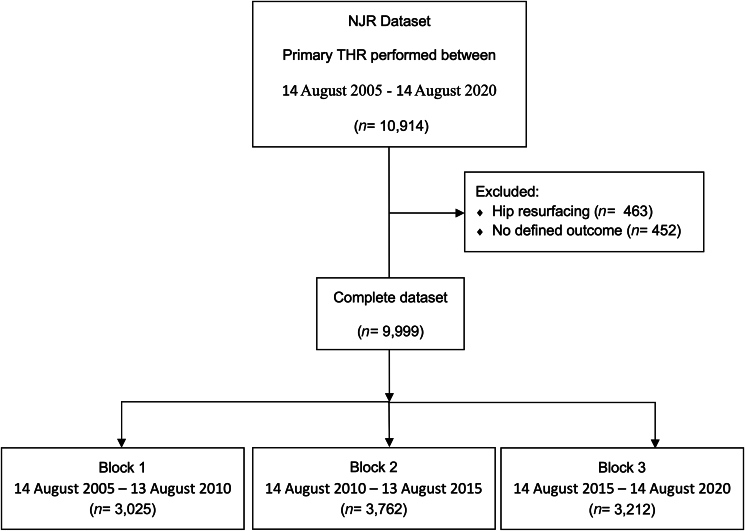
Flow diagram outlining case selection. NJR = National Joint Registry; THR = total hip replacement

This is a retrospective observational registry-based study. Institutional and NJR approval was granted to access data on patients who underwent elective primary THR in a single high-volume University Teaching Hospital from 2005 to 2020. The unique patient medical record number allowed linking of data from the generic NJR H1 (primary hip) and H2 (revision hip) forms. Patient outcomes in this dataset were categorised as unrevised, revised, mortality and unknown. Cases with unknown outcomes were removed prior to analysis (*n*=452). The one-year outcome for each patient was defined as the first event recorded within 365 days of primary elective THR. Data for primary hybrid, cemented and uncemented implant fixation combinations were included, with hip resurfacing operations excluded to avoid skewing revision outcomes (*n*=463).^5,6^ The lead operating surgeon grade was categorised as consultant-led (CL) or trainee-led (TL) for each case. Any grade of surgeon except consultant was grouped as a trainee. The first assistant grade is categorised as consultant or “other” on both NJR forms. Any TL operation with a consultant registered as first assistant was categorised as trainee-supervised (TS), and as trainee-unsupervised (TU) if performed without a consultant as first assistant. Patient age and body mass index (BMI) data were transformed from continuous into categorical variables using 20-year age ranges and standard BMI categories for reporting patient demographics, but were treated as continuous variables in the logistic regression models.^7^ Patient American Society of Anesthesiologists (ASA) physical status grades were reclassified into two groups for reporting demographics and in the logistic regression models.^8^ These groups represented healthy patients or those with mild systemic disease (ASA I/II), and those with severe systemic disease that may threaten life (ASA III/IV). Data analysis was performed on the 15-year dataset and split into 5-year blocks (B1 2005–2010, B2 2010–2015, B3 2015–2020) to determine any temporal trends in revision or mortality rates (Figure 1). A period of one year following primary THR was used as the threshold to determine early patient outcomes in this study. This threshold was selected because the modes of failure following THR differ dependent upon the interval from index procedure. Most early and potentially avoidable complications requiring revision surgery are identified shortly after the primary operation; for example, prosthetic joint infection (PJI) from direct inoculation at the time of surgery, or component malsizing/malpositioning leading to instability.^9,10^ The one-year threshold used in this study aims to differentiate these early and potentially avoidable reasons for revision from the late and potentially unavoidable revision operations (eg, polyethylene wear or periprosthetic fracture (PPF)). This threshold is supported by the results of a recent study, which reported a higher risk of implant failure within six months of TL primary THR.^1^

### Data analysis

Survival analyses were performed separately for one-year patient mortality or all-cause revision outcomes in relation to both lead surgeon grade (TL/CL) and the level of supervision (TS/TU).^11^ Kaplan–Meier survival estimate plots were used to visualise outcomes, and log-rank tests used to determine any significant difference in survival between the independent groups tested (lead surgeon grade and level of supervision).^12^ Multivariate logistic regression models were used to investigate factors associated with these outcome measures, including patient demographics (age, BMI, gender, ASA grade), and implant fixation as covariates. Descriptive statistics were used to report patient demographic data. Pearson's Chi-squared test was used to determine any significant differences observed between categorical variables. The significance threshold was set at 0.05 for all statistical analysis. Statistical analysis and graphical visualisation were performed using RStudio (RStudio Team (2023): Integrated Development for R. RStudio, PBC, Boston, MA).

## Results

A total of 9,999 primary THR met the eligibility criteria for this study. A total of 3,025 cases were recorded in B1 (30.3%); 3,762 in B2 (37.6%) and 3,212 in B3 (32.1%). Hybrid implant fixation combinations were the most used, representing 73.3% (*n*=7,334) of all cases. The mean patient age was 68.1 years (standard deviation (SD) 12.7), with the 61- to 80-year-old age category representing the largest overall with 62.4% (*n*=6,243) of patients. The mean patient BMI was 28.1kg/m^2^ (SD 5.6) with 38.3% (*n*=3,207) of patients falling in the overweight category (BMI 25–29.9kg/m^2^). No significant temporal changes were observed in patient BMI categories across the three five-year blocks. Female patients represented the highest percentage of patients at 61.2% overall, and no significant temporal changes were observed in patient sex (Table 1).

**Table 1:** Temporal changes in implant fixation, bearing size and patient demographics

	Overall, *N*=9,999*	B1: 2005–2010, *N*=3,025*	B2: 2010–2015, *N*=3,762*	B3: 2015–2020, *N*=3,212*	*p*-value^†^
Implant fixation					
Hybrid	7,334 (73.3%)	2,149 (71.0%)	2,806 (74.6%)	2,379 (74.1%)	**<0.001**
Cemented	1,496 (15.0%)	745 (24.6%)	502 (13.3%)	249 (7.8%)	
Uncemented	1,169 (11.7%)	131 (4.3%)	454 (12.1%)	584 (18.2%)	
Age, years					
<20	21 (0.2%)	7 (0.2%)	8 (0.2%)	6 (0.2%)	**<0.001**
21–40	345 (3.5%)	98 (3.2%)	127 (3.4%)	120 (3.7%)	
41–60	1,926 (19.3%)	510 (16.9%)	744 (19.8%)	672 (20.9%)	
61–80	6,243 (62.4%)	1,998 (66.0%)	2,364 (62.8%)	1,881 (58.6%)	
81–100	1,464 (14.6%)	412 (13.6%)	519 (13.8%)	533 (16.6%)	
BMI class					
Underweight	119 (1.4%)	18 (1.0%)	59 (1.7%)	42 (1.4%)	0.5
Normal	2,130 (25.4%)	447 (25.3%)	904 (25.6%)	779 (25.3%)	
Overweight	3,207 (38.3%)	710 (40.1%)	1,317 (37.3%)	1,180 (38.3%)	
Obese	2,667 (31.8%)	545 (30.8%)	1,139 (32.3%)	983 (31.9%)	
Morbidly Obese	252 (3.0%)	49 (2.8%)	110 (3.1%)	93 (3.0%)	
Unknown	1,624	1,256	233	135	
Sex					
Female	6,117 (61.2%)	1,877 (62.0%)	2,263 (60.2%)	1,977 (61.6%)	0.2
Male	3,882 (38.8%)	1,148 (38.0%)	1,499 (39.8%)	1,235 (38.4%)	
ASA					
1/2	7,782 (77.8%)	2,320 (76.7%)	3,003 (79.8%)	2,459 (76.6%)	**<0.001**
3/4	2,217 (22.2%)	705 (23.3%)	759 (20.2%)	753 (23.4%)	

ASA =  American Society of Anesthesiologists; BMI = body mass index

* *n* (%)

^†^Pearson's Chi-squared test; bold values indicate statistical significance

### Lead surgeon grade and level of supervision

Overall, 55.3% of primary THRs were performed by a consultant as lead surgeon (*n*=5,526), with significant temporal variation observed in the proportion performed by trainees (Table 2). Of the 44.7% of cases (*n*=4,473) performed by trainees, 46.3% were consultant supervised (Table 3). Significant temporal changes in the proportion of consultant supervised TL THRs were observed, with a gradual reduction in the proportion of unsupervised TL operations, from 62.4% in B1 (*n*=729/1,168) to 59.2% in B2 (*n*=1,051/1,775) and 40.8%, in B3 (*n*=624/1,530) (Table 3).

**Table 2:** Temporal changes in lead surgeon grade and level of supervision

	Overall, *N*=9,999*	B1: 2005–2010, *N*=3,025*	B2: 2010–2015, *N*=3,762*	B3: 2015–2020, *N*=3,212*	*p*-value^†^
Lead surgeon grade					
Consultant	5,526 (55.3%)	1,857 (61.4%)	1,987 (52.8%)	1,682 (52.4%)	**<0.001**
Trainee	4,473 (44.7%)	1,168 (38.6%)	1,775 (47.2%)	1,530 (47.6%)	
Level of supervision					
Consultant	5,526 (55.3%)	1,857 (61.4%)	1,987 (52.8%)	1,682 (52.4%)	**<0.001**
Supervised trainee	2,069 (20.7%)	439 (14.5%)	724 (19.2%)	906 (28.2%)	
Unsupervised trainee	2,404 (24.0%)	729 (24.1%)	1,051 (27.9%)	624 (19.4%)	

*n (%)

^†^Pearson's Chi-squared test; bold values indicate statistical significance

**Table 3:** Temporal changes in the level of supervision of TL THR

	Overall, *N*=4,473*	B1: 2005–2010, *N*=1,168*	B2: 2010–2015, *N*=1,775*	B3: 2015–2020, *N*=1,530*	*p*-value^†^
Level of supervision					
Supervised trainee	2,069 (46.3%)	439 (37.6%)	724 (40.8%)	906 (59.2%)	**<0.001**
Unsupervised trainee	2,404 (53.7%)	729 (62.4%)	1,051 (59.2%)	624 (40.8%)	

THR = total hip replacement; TL = trainee-led

*n (%)

^†^Pearson's Chi-squared test; bold values indicate statistical significance

### Incidence of one-year mortality and all-cause revision

The overall incidence of one-year mortality was 2.05% (*n*=205), and one-year all-cause revision was 1.11% (*n*=111). No difference was observed in the overall incidence of one-year mortality or all-cause revision with respect to the lead surgeon grade, or level of supervision (Table 4). The incidence of one-year all-cause revision differed significantly according to the patient BMI category, with 0.0% incidence observed in underweight, 0.6% in normal weight, 1.2% in both overweight and obese and 3.6% in morbidly obese patients (Table 4).

**Table 4:** Incidence of one-year patient mortality and all-cause revision, surgeon grade and supervision, implant fixation and patient demographics

	Mortality			Revision		
	Alive*	Mortality*	*p*-value^†^	Not revised*	Revised*	*p*-value^†^
Block						
2005–2010	2,939 (97.2%)	86 (2.8%)	**0.001**	2,992 (98.9%)	33 (1.1%)	0.2
2010–2015	3,698 (98.3%)	64 (1.7%)		3,728 (99.1%)	34 (0.9%)	
2015–2020	3,157 (98.3%)	55 (1.7%)		3,168 (98.6%)	44 (1.4%)	
Lead surgeon grade						
Consultant	5,403 (97.8%)	123 (2.2%)	0.2	5,471 (99.0%)	55 (1.0%)	0.2
Trainee	4,391 (98.2%)	82 (1.8%)		4,417 (98.7%)	56 (1.3%)	
Supervision						
Consultant	5,403 (97.8%)	123 (2.2%)	0.3	5,471 (99.0%)	55 (1.0%)	0.5
Supervised Trainee	2,028 (98.0%)	41 (2.0%)		2,043 (98.7%)	26 (1.3%)	
Unsupervised trainee	2,363 (98.3%)	41 (1.7%)		2,374 (98.8%)	30 (1.2%)	
Implant fixation						
Hybrid	7,231 (98.6%)	103 (1.4%)	**<0.001**	7,247 (98.8%)	87 (1.2%)	0.4
Cemented	1,401 (93.6%)	95 (6.4%)		1,484 (99.2%)	12 (0.8%)	
Uncemented	1,162 (99.4%)	7 (0.6%)		1,157 (99.0%)	12 (1.0%)	
Age						
<20	21 (100.0%)	0 (0.0%)	**<0.001**	20 (95.2%)	1 (4.8%)	0.2
21–40	340 (98.6%)	5 (1.4%)		342 (99.1%)	3 (0.9%)	
41–60	1,896 (98.4%)	30 (1.6%)		1,899 (98.6%)	27 (1.4%)	
61–80	6,140 (98.4%)	103 (1.6%)		6,181 (99.0%)	62 (1.0%)	
81–100	1,397 (95.4%)	67 (4.6%)		1,446 (98.8%)	18 (1.2%)	
BMI class						
Underweight	115 (96.6%)	4 (3.4%)	0.3	119 (100.0%)	0 (0.0%)	**0.001**
Normal	2,087 (98.0%)	43 (2.0%)		2,118 (99.4%)	12 (0.6%)	
Overweight	3,156 (98.4%)	51 (1.6%)		3,167 (98.8%)	40 (1.2%)	
Obese	2,629 (98.6%)	38 (1.4%)		2,636 (98.8%)	31 (1.2%)	
Morbidly Obese	248 (98.4%)	4 (1.6%)		243 (96.4%)	9 (3.6%)	
Unknown	1,559	65		1,605	19	
Sex						
Female	6,014 (98.3%)	103 (1.7%)	**0.001**	6,054 (99.0%)	63 (1.0%)	0.3
Male	3,780 (97.4%)	102 (2.6%)		3,834 (98.8%)	48 (1.2%)	
ASA						
1/2	7,709 (99.1%)	73 (0.9%)	**<0.001**	7,702 (99.0%)	80 (1.0%)	0.14
3/4	2,085 (94.0%)	132 (6.0%)		2,186 (98.6%)	31 (1.4%)	

ASA =  American Society of Anesthesiologists; BMI = body mass index

*n (%)

^†^Pearson's Chi-squared test; Fisher's exact test; bold values indicate statistical significance

### The impact of lead surgeon grade on one-year mortality and all-cause revision

#### Mortality

For CL operations the incidence of one-year patient mortality was 2.23% (*n*=123/5,526), in comparison with 1.83% (*n*=82/4,473) in TL operations (Table 4). No differences were observed in the incidence of one-year mortality between CL and TL operations overall or within any five-year block (Table 4).

#### Revision

**Figure 2:**
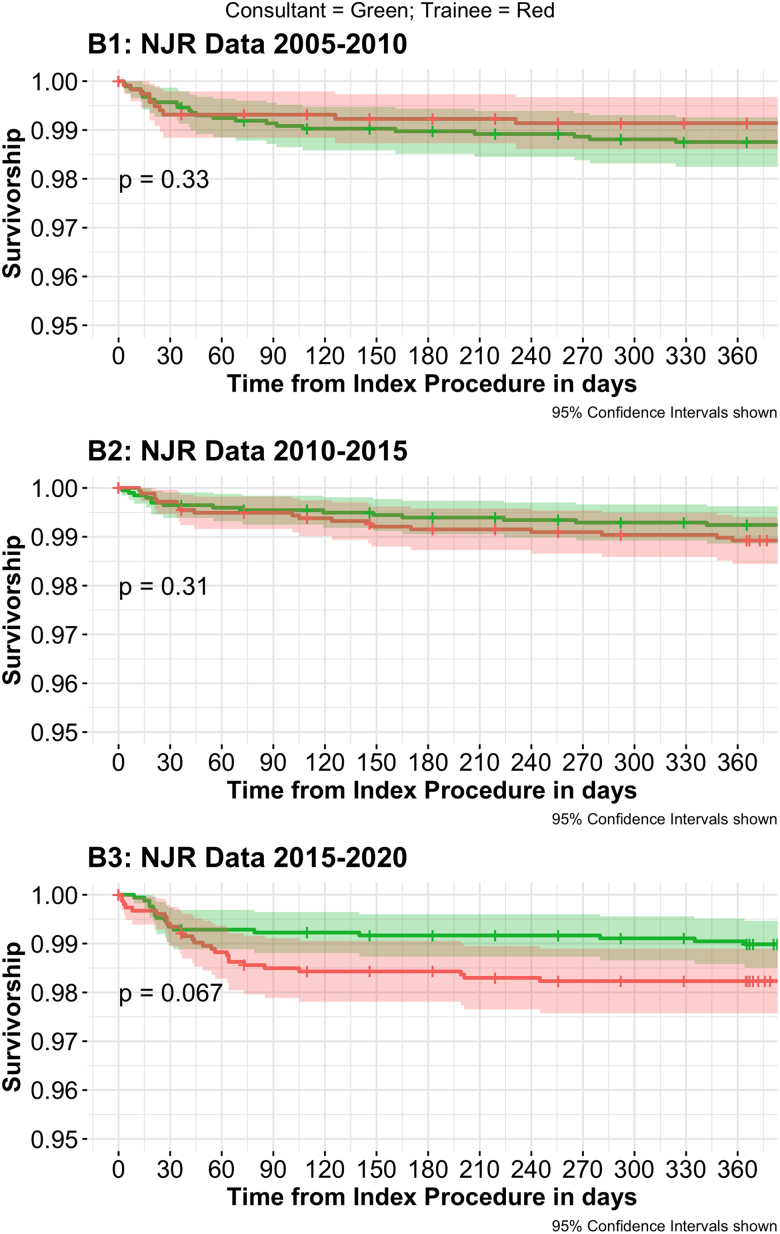
Kaplan­–Meier survival plots of one-year patient all-cause revision in relation to lead surgeon grade across the three five-year blocks. NJR = National Joint Registry

For CL operations, the incidence of one-year all-cause revision was 1.01% (*n*=55/5,526) in comparison with 1.27% (*n*=56/4,473) in TL operations (Table 4). No differences were observed in the incidence of one-year all-cause revision between CL and TL operations overall or within any five-year block (Table 4 and Figure 2).

### The impact of trainee supervision on one-year mortality and all-cause revision

#### Mortality

For TS operations, the incidence of one-year patient mortality was 1.98% (*n*=41/2,069), in comparison with 1.71% (*n*=41/2,404) in TU operations (Table 4). No differences were observed in the incidence of one-year patient mortality between consultant and TS or TU operations overall or within any five-year block (Table 4).

#### Revision

**Figure 3:**
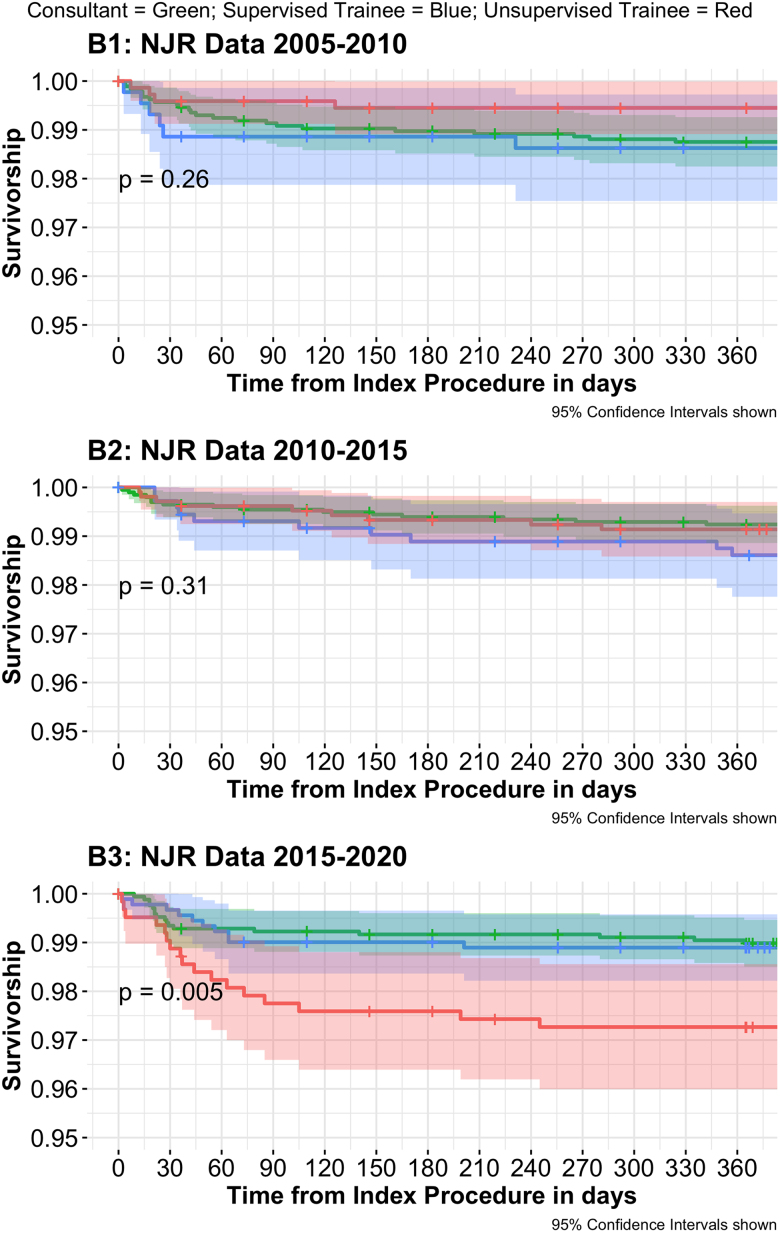
Kaplan–Meier survival plots of one-year patient all-cause revision in relation to the level of supervision across the three 5-year blocks. NJR = National Joint Registry

For TS operations, the overall incidence of one-year all-cause revision was 1.26% (*n*=26/2,069) in comparison with 1.25% (*n*=30/2,404) in TU operations (Table 4). No differences were observed in the overall incidence of one-year patient all-cause revision between TS and TU operations in comparison with CL operations. However, a significantly increased incidence of one-year all-cause revision was identified between TU and CL operations in B3 data from 2015 to 2020 (odds ratio (OR)=2.81, 95% confidence interval (CI) 1.35–5.87, *p*=0.005) (Tables 5 and 6, and Figure 3). The incidence of one-year all-cause revision in TU operations from 2015 to 2020 was 2.72% (*n*=17/624), in comparison with 1.1% (*n*=10/906) in TS and 1.01% (*n*=17/1,682) in CL operations. The incidence of one-year all-cause revision in TU operations from 2005 to 2010 was 0.55% (*n*=4/729), and 0.86% (*n*=9/1,051) from 2010 to 2015. No significant differences were identified in the incidence of one-year all-cause revision between CL and TS operations within any of the five-year blocks.

**Table 5:** Incidence of one-year all-cause revision following primary THR in relation to the surgeon grade and level of trainee supervision

	Overall*	Consultant*	Supervised Trainee*	Unsupervised Trainee*	*p*-value^†^
Summary: 2005–2020					
Not Revised	9,888 (98.9%)	5,471 (99.0%)	2,043 (98.7%)	2,374 (98.8%)	0.5
Revised	111 (1.1%)	55 (1.0%)	26 (1.3%)	30 (1.2%)	
B1: 2005–2010					
Not revised	2,992 (98.9%)	1,834 (98.8%)	433 (98.6%)	725 (99.5%)	0.2
Revised	33 (1.1%)	23 (1.2%)	6 (1.4%)	4 (0.5%)	
B2: 2010–2015					
Not revised	3,728 (99.1%)	1,972 (99.2%)	714 (98.6%)	1,042 (99.1%)	0.3
Revised	34 (0.9%)	15 (0.8%)	10 (1.4%)	9 (0.9%)	
B3: 2015–2020					
Not revised	3,168 (98.6%)	1,665 (99.0%)	896 (98.9%)	607 (97.3%)	**0.005**
Revised	44 (1.4%)	17 (1.0%)	10 (1.1%)	17 (2.7%)	

THR = total hip replacement

*n (%)

^†^Pearson's Chi-squared test; bold values indicate statistical significance

### Indications for revision within one year

The indications for early revision were identified, namely dislocation/instability, PPF and PJI.^13^ Revisions undertaken for any other indication have been grouped as “other”. Overall, the most common indication for revision procedures within one year of primary THR was PJI at 31.5% (*n*=35/111), followed by dislocation/instability at 29.7% (*n*=33/111), all “other” indications at 20.7% (*n*=23/111) and PPF at 18.0% (*n*=20/111) (Table 7). No significant differences were observed in the indication for revision with respect to lead surgeon grade or level of supervision. A statistically significant difference was found in the indication for revision according to the level of supervision for B3 data (*p*=0.034, *n*=17). This appears to be driven by increased incidence of PJI (*n*=7/17) and dislocation/instability (*n*=7/17) in TU operations, which collectively represented the indication for revision in 82.4% of cases in this subgroup (*n*=14/17).

**Table 6:** Multivariate logistic regression results for one-year patient all-cause revision following primary THR in relation to the level of supervision

	Overall: 2005–2020			B1: 2005–2010			B2: 2010–2015			B3: 2015–2020		
	OR*	95% CI	*p*-value	OR*	95% CI	*p*-value	OR	95% CI	*p*-value	OR*	95% CI	*p*-value*
Surgeon combination												
Consultant	–	–		–	–		–	–		–	–	
Supervised trainee	1.39	0.82, 2.30	0.2	1.60	0.51, 4.26	0.4	1.85	0.72, 4.47	0.2	1.19	0.51, 2.67	0.7
Unsupervised trainee	1.35	0.80, 2.22	0.3	0.17	0.01, 0.91	0.10	1.28	0.49, 3.11	0.6	2.81	1.35, 5.87	**0.005**

CI = confidence interval; OR = odds ratio; THR = total hip replacement

^†^Bold values indicate statistical significance

**Table 7:** Indication for undergoing revision within one year following primary THR

	Overall*	Consultant*	Supervised Trainee*	Unsupervised Trainee*	*p*-value^†^
Summary: 2005–2020					
Not Revised	9,888 (98.9%)	5,471 (99.0%)	2,043 (98.7%)	2,374 (98.8%)	0.6
Infection	35 (0.4%)	18 (0.3%)	7 (0.3%)	10 (0.4%)	
Dislocation/instability	33 (0.3%)	16 (0.3%)	8 (0.4%)	9 (0.4%)	
PPF	20 (0.2%)	8 (0.1%)	4 (0.2%)	8 (0.3%)	
Other	23 (0.2%)	13 (0.2%)	7 (0.3%)	3 (0.1%)	
B1: 2005–2010					
Not revised	2,992 (98.9%)	1,834 (98.8%)	433 (98.6%)	725 (99.5%)	0.9
Infection	8 (0.3%)	5 (0.3%)	2 (0.5%)	1 (0.1%)	
Dislocation/instability	14 (0.5%)	11 (0.6%)	2 (0.5%)	1 (0.1%)	
PPF	5 (0.2%)	3 (0.2%)	1 (0.2%)	1 (0.1%)	
Other	6 (0.2%)	4 (0.2%)	1 (0.2%)	1 (0.1%)	
B2: 2010–2015					
Not revised	3,728 (99.1%)	1,972 (99.2%)	714 (98.6%)	1,042 (99.1%)	0.4
Infection	6 (0.2%)	3 (0.2%)	1 (0.1%)	2 (0.2%)	
Dislocation/instability	7 (0.2%)	3 (0.2%)	3 (0.4%)	1 (0.1%)	
PPF	10 (0.3%)	3 (0.2%)	2 (0.3%)	5 (0.5%)	
Other	11 (0.3%)	6 (0.3%)	4 (0.6%)	1 (0.1%)	
B3: 2015–2020					
Not revised	3,168 (98.6%)	1,665 (99.0%)	896 (98.9%)	607 (97.3%)	**0.033**
Infection	21 (0.7%)	10 (0.6%)	4 (0.4%)	7 (1.1%)	
Dislocation/instability	12 (0.4%)	2 (0.1%)	3 (0.3%)	7 (1.1%)	
PPF	5 (0.2%)	2 (0.1%)	1 (0.1%)	2 (0.3%)	
Other	6 (0.2%)	3 (0.2%)	2 (0.2%)	1 (0.2%)	

PPF = periprosthetic fracture; THR = total hip replacement

*n (%)

^†^Pearson's Chi-squared test; bold values indicate statistical significance

## Discussion

One of the most striking findings of this study is the significant temporal increase in the incidence of revision procedures within one year of primary THR operations performed by unsupervised trainees in comparison with either supervised trainees or consultants. The indication for these early revisions appears to be driven predominantly by an increased incidence of PJI and dislocation/instability, which are recognised common indications for early revision.^13–16^ These findings are supported by the results of another recent observational study using NJR data.^1^ The aetiology of these early revisions cannot be determined using this NJR dataset alone, but the absence of consultant supervision appears to be an important factor. Patients who undergo early revision procedures are reported to have poorer long-term outcomes, and an increased risk of re-revision procedures.^14,17–21^ This highlights the importance of Getting It Right First Time to reduce complications, healthcare costs and optimise patient outcomes following elective orthopaedic surgery.^22^ Furthermore, any revision procedures will be recorded under the lead consultant NJR figures, which may place them at increased risk of falling into outlier status. Interestingly, the increased incidence of early revisions in unsupervised TL operations was not seen over the entire 15-year data collection period. This suggests that the increased incidence of early revisions in unsupervised TL THR is a relatively recent phenomenon. Over the 15-year data period, significant temporal increases have been observed in the proportion of primary THR performed by trainees (*p*<0.001), but with a gradual reduction in the proportion of unsupervised operations, from 62.4% in B1 (2005–2010) to 40.8% in B3 (2015–2020). One potential explanation may be that, in recent years, trainees have less operative experience than their predecessors, which may affect their intraoperative performance, judgement, and the incidence of postoperative adverse patient outcomes. The reduction in proportion of TL procedures being unsupervised may also indicate the growing awareness among consultant trainers that fewer trainees are able to perform THR unsupervised, or reach that stage only later in their training. The findings of this study therefore have wide-ranging implications not only for patients, trainee surgeons and NJR consultant profiles, but for those responsible for designing training programmes to maintain a competent surgical workforce for the future.

As a large-volume tertiary referral centre, there is a strong tendency for trainees in the hip team to be post-CCT fellows, rather than specialty registrars (three fellows and one registrar per six-month period). This makes these results concerning, as performing THR independently is an important stage in the trainee’s learning curve. This highlights a potential training issue, that the consultant trainers are familiar with “finessing” a senior trainee who has already benefitted from training elsewhere and has reached, or almost reached, a level of competence to operate independently, but have poorer skills in providing training for a less experienced trainee. With the change in training and later years’ trainees being less experienced, this effect may be amplified more recently. Our unit has a track record in preparing the senior trainees for their consultant posts, evidenced by the exceptionally high proportion of TL operations (44.7%) when compared with the recently reported proportion across the whole of the NJR (9.6%).^1^ This has implications for individual consultant surgeons who may feel that this proportion of TL procedures is unsustainable with the current model of outlier status calculation by the NJR. This latter topic of outlier analysis (ie, a consultant surgeon’s results compared with their peers) is important to many consultant surgical trainers. The NJR might wish to consider the findings and implications of this study with respect to consultant surgeons who have a recognised role in education and training, particularly in relation to setting thresholds for determining outlier status and the acceptable incidence of revision procedures following primary THR.

A further explanation for the observed increase in unsupervised TL revisions may be related to the experience of trainers. Where there is a significant learning curve to perform a procedure, which continues after appointment as a consultant, there is also a significant learning curve on how to train a procedure. A surgeon who has supervised many trainees of varying degrees of seniority may be a better judge of when a trainee is competent to operate independently than a supervising surgeon with less experience. This effect may be amplified since more recently appointed consultants are likely to have performed fewer THRs during their training than in previous years. Conversely, newly appointed consultants may be more cautious, and have a higher threshold for allowing surgeons to operate independently than those with more experience. This uncertainty highlights the need to exercise caution on how these results are interpreted. If experienced trainers stop senior trainees performing THRs unsupervised in response to these data, newly appointed consultants will be less far along their learning curve for performing the procedure. The risk of providing patients with suboptimal care, and potentially becoming an NJR outlier, is a source of great anxiety for surgeons.

It is reassuring to report that no significant differences were identified in the risk of one-year mortality or all-cause revision between CL, overall TL or trainee-supervised operations across the entire 15-year data collection period, or within any of the 5-year blocks. This finding supports those of other published studies that have reported no significantly increased risk of revision procedures in patients undergoing THR operations performed by trainees in comparison with consultants.^1,2,23–26^ The results of one systematic review and meta-analysis reported no significant difference in revision rates between TL and CL primary THR (OR 1.09, *p*=0.51).^2^ However, the time to revision and level of supervision for TL operations were not considered in this meta-analysis. Another recent systematic review and meta-analysis of five eligible studies analysed a total of 1,464 TL THR and 2,602 CL THR over a 10-year follow-up period.^23^ The authors reported a relative risk of revision for TL primary THR of 0.88 (95% CI 0.46–1.70) at five years, and 0.68 (95% CI 0.37 to 1.26) at ten years, respectively. A recent large observational study using an NJR dataset of 603,474 primary THR reported no association between surgeon grade and all-cause revision up to ten years, with a hazard ratio of 1.00 (95% CI 0.04–1.07, *p*=0.966).^1^ However, in this latter study only 9.6% of all primary THR were recorded as TL (*n*=58,137).

The provision of high-quality training is critically important to ensuring a highly skilled future surgical workforce, and the results of this study should not be used to reduce surgical training opportunities. The results should be used as a prompt for surgical trainers and units to consider the theatre environment, human resources, surgical equipment, and patient case selection that are best suited for providing the optimal surgical training opportunities. Surgical trainees should be supported and encouraged by trainers and healthcare institutions to operate within the limits of their competence and always with appropriate educational and clinical supervision. Postgraduate medical education and training boards should strongly consider alternative strategies and methods for supporting trainees' learning and development outside of the immediate theatre environment. This may include an increased provision of evidence-based surgical simulation training to assist trainees in acquiring, optimising and transferring their technical surgical skills performance in the safety of a nonclinical environment prior to refining them in theatre under the direct supervision of experienced consultant trainers.^27,28^ Mechanisms to identify and address adverse patient outcomes and complications should be embodied within the clinical governance structure of all healthcare institutions. Departmental morbidity and mortality meetings are one example of a forum where all staff should be encouraged to openly discuss complications without apportioning blame, investigate potential causative factors and implement positive changes aimed at improving patient care and outcomes. Self-reflection is also an important aspect of continuing professional development, which all surgeons should engage in to identify potential areas for personal improvement.

The response to these results in our department was to identify a strategy to allow senior trainees to progress to independent operating while minimising the risk of adverse outcomes. Analysis of the patient risk factors showed that patients between 70 and 80 years, with primary osteoarthritis and a BMI less than 35kg/m^2^, who underwent hybrid THR fixation had the lowest risk of revision in the unsupervised TL cohort. Therefore, independent trainee lists are populated with patients who satisfy these criteria. In addition, all patients are discussed preoperatively with the overseeing consultant to identify potential challenges, such as abnormal morphology or human factors.

A strength of this study is that the demographics of patients undergoing primary THR in this dataset are broadly representative of patients across the UK as reported in the NJR,^29^ with 61.2% female patients (59.9% in the NJR), a median average age of 70 years (median 69 in the NJR), and 77.8% ASA I/II (83.2% in the NJR). The main limitation of this study is the methodology for categorising surgeons. Any lead surgeon who was not recorded on the NJR H1 form as a consultant was categorised as a trainee. This included both specialty doctors and associate specialists who usually possess higher levels of surgical experience than trainees. However, as only 0.6% of all cases (*n*=56) were performed by surgeons in these grades, it is unlikely to impact the main findings of this study. The trainee cohort therefore contained registrars across all years and post-CCT fellows and we did not perform further analysis by level of trainee. Given the retrospective observational nature of this study it was also impossible to account for individual case complexity. However, it is reasonable and pragmatic to assume that the cases performed by trainees were reviewed appropriately and selected preoperatively by the supervising consultant surgeon. Furthermore, as with every registry-based study, the quality of data inputted directly influences the validity of the results generated. Incorrectly coded or missing data, along with changes in the scope of data collected over time may result in inaccurate interpretations and conclusions.^30,31^ For example, the overall compliance in recording of patient BMI data in this study was 83.76%, but this figure has improved progressively over time from 58.48% cases in block 1 to 93.8% in block 2 and 95.8% in block 3. It is reassuring that compliance in recording patient age, gender, ASA and implant fixation as well as the lead operating surgeon and assistant grade was 100% throughout the entire study period. It is also noteworthy that the NJR H2 forms will not capture all revision hip procedures, especially for cases of early instability or fracture, where closed manipulation/s or open reduction and internal fixation procedures, respectively, may be performed.^32^ Future studies with linked Hospital Episode Statistic data may be more accurate in defining the true incidence of revision procedures in the early- to mid-term postoperative period.

In conclusion, this study has highlighted the importance of appropriate consultant supervision during primary THR performed by surgical trainees which may, in conjunction with other reported risk factors, reduce the incidence of potentially avoidable early revision operations.^33,34^ This finding has important implications for trainees, trainers, healthcare institutions and those responsible for building the future healthcare workforce.

## References

[1] Fowler TJ, Aquilina AL, Reed MR *et al.* The association between surgeon grade and risk of revision following total hip arthroplasty. *Bone Joint J* 2022; **104-B**: 341–351. 35227094 10.1302/0301-620X.104B3.BJJ-2021-1389.R1

[2] Singh P, Madanipour S, Fontalis A *et al.* A systematic review and meta-analysis of trainee- versus consultant surgeon-performed elective total hip arthroplasty. *EFORT Open Rev* 2019; **4**: 44–55. 30931148 10.1302/2058-5241.4.180034 PMC6404792

[3] Schoenfeld AJ, Serrano JA, Waterman BR *et al.* The impact of resident involvement on post-operative morbidity and mortality following orthopaedic procedures: a study of 43,343 cases. *Arch Orthop Trauma Surg* 2013; **133**: 1483–1491. 23995548 10.1007/s00402-013-1841-3

[4] Pugely AJ, Gao Y, Martin CT *et al.* The effect of resident participation on short-term outcomes after orthopaedic surgery. *Clin Orthop Relat Res* 2014; **472**: 2290–2300. 24658902 10.1007/s11999-014-3567-0 PMC4048420

[5] Prosser GH, Yates PJ, Wood DJ *et al.* Outcome of primary resurfacing hip replacement: evaluation of risk factors for early revision. *Acta Orthop* 2010; **81**: 66–71. 20180719 10.3109/17453671003685434 PMC2856206

[6] Marshall DA, Pykerman K, Werle J *et al.* Hip resurfacing versus total hip arthroplasty: a systematic review comparing standardized outcomes. *Clin Orthop Relat Res* 2014; **472**: 2217–2230. 24700446 10.1007/s11999-014-3556-3 PMC4048407

[7] Nuttall FQ. Body mass index: obesity, BMI, and health: a critical review. *Nutr Today* 2015; **50**: 117–128. 27340299 10.1097/NT.0000000000000092 PMC4890841

[8] Doyle DJ, Goyal A, Bansal P *et al.* *American Society of Anesthesiologists Classification*. Treasure Island, FL: StatPearls; 2022.

[9] Novikov D, Mercuri JJ, Schwarzkopf R *et al.* Can some early revision total hip arthroplasties be avoided? *Bone Joint J* 2019; **101-B**: 97–103. 31146556 10.1302/0301-620X.101B6.BJJ-2018-1448.R1

[10] Karachalios T, Komnos G, Koutalos A. Total hip arthroplasty: survival and modes of failure. *EFORT Open Rev* 2018; **3**: 232–239. 29951261 10.1302/2058-5241.3.170068 PMC5994632

[11] Therneau TM. *A package for survival analysis in R. R package version 3.7-0*. https://CRAN.R-project.org/package=survival (cited September 2023).

[12] Bland JM, Altman DG. The logrank test. *BMJ* 2004; **328**: 1073. 15117797 10.1136/bmj.328.7447.1073 PMC403858

[13] Duman S, Çamurcu İY, Uçpunar H *et al.* Comparison of clinical characteristics and 10-year survival rates of revision hip arthroplasties among revision time groups. *Arch Med Sci* 2021; **17**: 382–389. 33747274 10.5114/aoms.2019.88563 PMC7959053

[14] Roof MA, Yeroushalmi D, Aggarwal V *et al.* Time to revision hip arthroplasty (rTHA) impacts outcomes following rTHA. *Orthop Proc* 2020; **102-B**: 35.

[15] Heckmann ND, Yang J, Ong KL *et al.* Revision surgery for instability after total hip arthroplasty: does timing matter? *J Arthroplasty* 2021; **36**: 1779–1783.e2. 33504458 10.1016/j.arth.2020.12.035

[16] Sabah SA, Knight R, Nicolson PJA *et al.* Epidemiology of revision hip replacement surgery in the UK over the past 15 years—an analysis from the national joint registry. *BMJ Open* 2023; **13**: e072462. 10.1136/bmjopen-2023-072462 PMC10583040 37848303

[17] Khatod M, Cafri G, Inacio MC *et al.* Revision total hip arthoplasty: factors associated with re-revision surgery. *J Bone Joint Surg Am* 2015; **97**: 359–366. 25740025 10.2106/JBJS.N.00073

[18] Harada S, Hamai S, Shiomoto K *et al.* Patient-reported outcomes after primary or revision total hip arthroplasty: a propensity score-matched Asian cohort study. *PLoS ONE* 2021; **16**: e0252112. 34043675 10.1371/journal.pone.0252112 PMC8158935

[19] Postler AE, Beyer F, Wegner T *et al.* Patient-reported outcomes after revision surgery compared to primary total hip arthroplasty. *Hip Int* 2017; **27**: 180–186. 27886353 10.5301/hipint.5000436

[20] Yu S, Saleh H, Bolz N *et al.* Re-revision total hip arthroplasty: epidemiology and factors associated with outcomes. *J Clin Orthop Trauma* 2020; **11**: 43–46. 32001983 10.1016/j.jcot.2018.08.021 PMC6985171

[21] Hermansen LL, Viberg B, Overgaard S. Risk factors for dislocation and re-revision after first-time revision total hip arthroplasty due to recurrent dislocation—a study from the Danish hip arthroplasty register. *J Arthroplasty* 2021; **36**: 1407–1412. 33423877 10.1016/j.arth.2020.10.004

[22] Barratt H, Turner S, Hutchings A *et al.* Mixed methods evaluation of the Getting it Right First Time programme—improvements to NHS orthopaedic care in England: study protocol. *BMC Health Serv Res* 2017; **17**: 71. 28115018 10.1186/s12913-017-2012-y PMC5260012

[23] Fowler TJ, Aquilina AL, Blom AW *et al.* Association between surgeon grade and implant survival following hip and knee replacement: a systematic review and meta-analysis. *BMJ Open* 2021; **11**: e047882. 10.1136/bmjopen-2020-047882 PMC8587578 34758989

[24] Bron DM, Wolterbeek N, Poolman RW *et al.* Resident training does not influence the complication risk in total knee and hip arthroplasty. *Acta Orthop* 2021; **92**: 689–694. 34605337 10.1080/17453674.2021.1979296 PMC8635675

[25] Inglis T, Dalzell K, Hooper G *et al.* Does orthopedic training compromise the outcome in total hip arthroplasty? *J Surg Educ* 2013; **70**: 76–80. 23337674 10.1016/j.jsurg.2012.08.003

[26] Wilson MD, Dowsey MM, Spelman T *et al.* Impact of surgical experience on outcomes in total joint arthroplasties. *ANZ J Surg* 2016; **86**: 967–972. 27598857 10.1111/ans.13513

[27] Kalun P, Wagner N, Yan J *et al.* Surgical simulation training in orthopedics: current insights. *Adv Med Educ Pract* 2018; **9**: 125–131. 29503591 10.2147/AMEP.S138758 PMC5826303

[28] Howgate D, Garfjeld Roberts P, Kendrick B *et al.* Key performance and training parameters in primary total hip arthroplasty - an expert consensus using the Delphi technique. *Hip Int* 2023; **33**: 411–419. 34748447 10.1177/11207000211056864 PMC10170576

[29] Ben-Shlomo Y, Blom A, Boulton C *et al.* *The National Joint Registry 18th Annual Report 2021*. London: National Joint Registry; 2021. https://www.ncbi.nlm.nih.gov/books/NBK576858/ (cited September 2023). 35072993

[30] Boulton C, Harrison C, Wilton T *et al.* Implementing large-scale data quality validation in a national arthroplasty registry to improve compliance: the National Joint Registry data quality audit programme. *Bone Jt Open* 2022; **3**: 716–725. 36106695 10.1302/2633-1462.39.BJO-2022-0051.R1 PMC9533243

[31] Afzal I, Radha S, Smoljanović T *et al.* Validation of revision data for total hip and knee replacements undertaken at a high volume orthopaedic centre against data held on the National Joint Registry. *J Orthop Surg Res* 2019; **14**: 318. 31601231 10.1186/s13018-019-1304-9 PMC6785883

[32] Bottle A, Griffiths R, White S *et al.* Periprosthetic fractures: the next fragility fracture epidemic? A national observational study. *BMJ Open* 2020; **10**: e042371. 10.1136/bmjopen-2020-042371 PMC7733197 33303466

[33] Bottle A, Parikh S, Aylin P, Loeffler M. Risk factors for early revision after total hip and knee arthroplasty: national observational study from a surgeon and population perspective. *PLoS ONE* 2019; **14**: e0214855. 30964880 10.1371/journal.pone.0214855 PMC6456180

[34] Dy CJ, Bozic KJ, Pan TJ *et al.* Risk factors for early revision after total hip arthroplasty. *Arthritis Care Res (Hoboken)* 2014; **66**: 907–915. 24285406 10.1002/acr.22240 PMC4269321

